# Mobile Biomonitoring of Atmospheric Pollution: A New Perspective for the Moss-Bag Approach

**DOI:** 10.3390/plants10112384

**Published:** 2021-11-05

**Authors:** Maria Cristina Sorrentino, Fiore Capozzi, Karen Wuyts, Steven Joosen, Valentine K. Mubiana, Simonetta Giordano, Roeland Samson, Valeria Spagnuolo

**Affiliations:** 1 Department of Biology, Campus Monte S. Angelo, University of Naples Federico II, Via Cinthia 4, 80126 Napoli, Italy; mariacristina.sorrentino@unina.it (M.C.S.); fiore.capozzi@unina.it (F.C.); giordano@unina.it (S.G.); 2 Department of Bioscience Engineering, Campus Groenenborgerlaan 171, University of Antwerp, 2020 Antwerp, Belgium; karen.wuyts@uantwerpen.be (K.W.); roeland.samson@uantwerpen.be (R.S.); 3 Department of Biology, Campus Groenenborgerlaan 171, University of Antwerp, 2020 Antwerp, Belgium; steven.joosen@uantwerpen.be (S.J.); kayawevalentine.mubiana@uantwerpen.be (V.K.M.)

**Keywords:** *Hypnum cupressiforme*, air biomonitoring, elemental pollution, SIRM

## Abstract

In this work the potential of moving moss-bags, fixed to bicycles, to intercept particulate matter (PM) and linked metal(loid)s was tested for the first time. Seven volunteers carried three moss-bags for fifty days while commuting by bicycle in the urban area of Antwerp, Belgium. Moreover, one bike, equipped with mobile PM samplers, travelled along four routes: urban, industrial, green route and the total path, carrying three moss-bags at each route. The saturation isothermal remanent magnetization (SIRM) signal and chemical composition (assessed by HR-ICP-MS) of the moss samples indicated that the industrial route was the most polluted. Element fluxes (i.e., the ratio between element daily uptake and the specific leaf area) could discriminate among land uses; particularly, they were significantly higher in the industrial route for Ag, As, Cd and Pb; significantly lowest in the green route for As and Pb; and comparable for all accumulated elements along most urban routes. A comparison with a previous experiment carried out in the same study area using similar moss-bags at static exposure points, showed that the element fluxes were significantly higher in the mobile system. Finally, PM2.5 and PM10 masses measured along the four routes were consistent with element fluxes.

## 1. Introduction

Air pollution is defined as the presence of harmful or poisonous substances in the Earth’s atmosphere, causing adverse effects on human health and ecosystems [1]. In fact, air pollution also affects animals, plants, and ecological resources including water and soils [2,3]. The WHO global conference on air pollution and health reported that over 4 million people died of air pollution every year and that about 91% of the world’s population live in places exceeding the thresholds reported by the WHO air quality guidelines. These latter fixed the limits for 24-h exposure to PM_2.5_ and PM_10_ at 25 and 50 μg m^−3^, respectively, but limits imposed by the national legislations are indeed more tolerant [4]. A wide body of literature have demonstrated that PM can determine the onset of diseases regardless its chemical composition, but also as carrier of toxicants and pathogens [5,6,7]. Especially in urban areas, air pollution levels undergo fluctuations in space and time due to the coexistence of different pollution sources and their constant or intermittent emissions; therefore, the evaluation of air quality is intrinsically complicated. Since the measurements of PM concentration directly in the atmosphere (i.e., through automatic devices) is very expensive and practically impossible at high spatial resolution [8], many researchers use biomonitors to detect airborne pollutants, and particularly PM [9,10,11]. Due to their intrinsic properties (e.g., sessile organisms, wide surface exposed to atmosphere) plants are particularly suited as biomonitors of airborne pollutants. Accordingly, recent literature demonstrated that the elemental contents measured in mosses transplanted in bags is significantly correlated with amounts of PM entrapped [12].

In biomonitoring, chemical analysis of the plant tissues can be profitably integrated with magnetic analysis, since these latter are sample-conservative, and require low costs and less time. Enviro-magnetic analysis of atmospheric PM by SIRM (saturation isothermal remanent magnetization) was employed since the 80s [13], but only in the last decades, this approach revealed its efficacy for monitoring air quality through the analysis of soil, sediments and road dust [14]. Since many plants are able to intercept and retain PM, several authors in recent years have studied the magnetic properties of vascular plant leaves and mosses to predict the level of metal pollution in the atmosphere [15,16]. This approach also provided useful information in biomonitoring of indoor environments by moss-bags [17].

Moss and lichen-bags have been valuably used in biomonitoring of air quality [9,12,16], but while their efficacy in bioaccumulation of pollutants (e.g., metals, metalloids, PAHs) is well known when they are exposed in open air and fixed positions, poor data exist on their potential ability in other exposure conditions. For example, few data are available on pollutant bioaccumulation by bags exposed indoors [17,18,19,20] and, to our knowledge, no data have been published so far on moss-bags exposed in mobile systems. These data are of fundamental importance for a global evaluation of the risk level related to the exposure to airborne pollutants. In many cities people reach their workplace or school by bikes or motorcycles to escape vehicular traffic and overcrowded public transport. Over the past year, due to the Covid 19 pandemic, we have seen a surge in the sale of bicycles, pedal assisted electric bikes, electric mopeds, and scooters to avoid the crowded wagons of the subway.

Based on all the above reported, the aim and novelty of this work was to test the potential of moss-bags fixed to bicycles to intercept airborne metal(loid)s. Given their capacity to discriminate pollution levels between relatively close areas [21], we hypothesized that moss-bags can be profitably used for the biomonitoring of air quality in moving systems; therefore, in this pilot work we aimed to provide answers to the following questions:

(i) Can the “mobile” moss-bags accumulate airborne elements and what are, if any, the criticisms related to this new approach?
(ii) Can the mobile moss-bags discriminate the different land uses of the investigated routes in term of pollution levels?
(iii) What is the relationship between data from mobile and static biomonitoring systems applied in the same study area?

The results are discussed in a methodological way to provide the first guidelines for this new approach.

## 2. Results

### 2.1. SIRM Analysis

**Figure 1:**
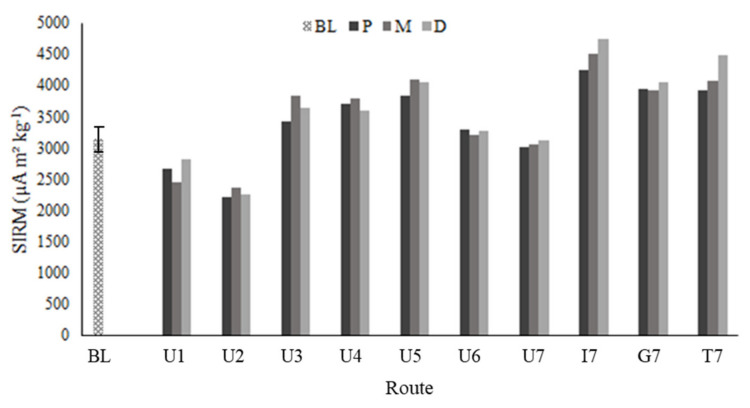
Saturation isothermal remanent magnetization (SIRM) value (µA m^2^ kg^−1^) of the unexposed moss (BL) and of the proximal (P), middle (M), and distal (D) moss-bags exposed along the cycling routes (see text for explanation of route codes).

The SIRM value of the unexposed moss was 3141 µA m^2^ kg^−1^ ± 183. The SIRM values of the exposed mosses ranged between 2283 µA m^2^ kg^−1^ (lower than unexposed moss) recorded at U2 route, and 4498 µA m^2^ kg^−1^ measured at 7I route (Figure 1).

**Figure 2:**
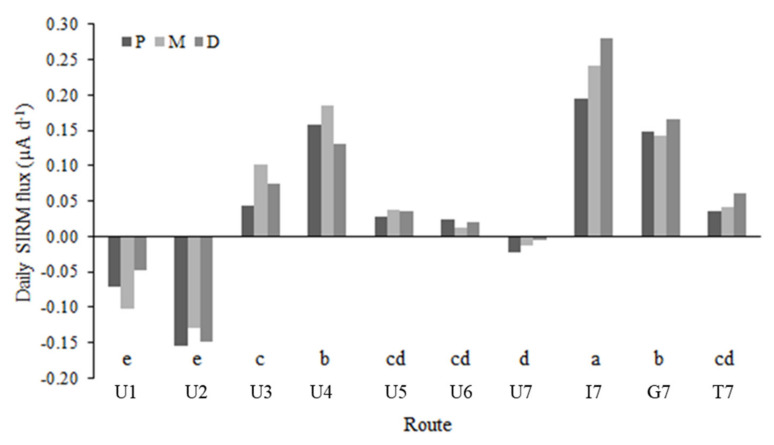
Daily SIRM flux (µA d^−1^) of the exposed moss in the proximal (P), middle (M), and distal (D) bags, calculated as the SIRM of the exposed moss minus the SIRM value of the unexposed moss and normalized by the exposure time and specific leaf area (SLA), for each route (see text for explanation of route codes). Different letters indicate significant (*p* < 0.05) differences between routes.

The SIRM data were always consistent in the three moss replicas exposed at each route. The daily SIRM flux, i.e., the SIRM values corrected for the mean SIRM value of the unexposed moss and normalized by the exposure time (Figure 2) was positive at all routes apart from U1, U2 and U7, with the highest value recorded at I7. Even between the routes in the same urban land use class, a lot of variation can be observed in the average daily SIRM fluxes. An ANOVA on the daily SIRM flux indicated a significant difference (*p* < 0.05) among the magnetic signals measured in the different land uses with I > G > U. The mean daily SIRM flux was 0.038, 0.052 and 0.057 µA d^−1^ for the P, M and D bags, respectively, but bag position did not significantly contribute to the explanation of the variation in net daily SIRM accumulation (*p* = 0.146). A positive, significant correlation was found between the SIRM signal and some elements (Table 1), including the magnetizable Co and Ni, with the highest correlation coefficient for Pb.

### 2.2. Chemical Analysis: Element Accumulation and Flux

Apart from the route T7, including U7, I7, 7G, and connecting paths, where the moss-bags were exposed for the longest time, several elements of environmental concern (As, Cd, Ni and Pb) showed the highest concentrations in I7 (Tables S1 and S2). However, because of the high overlap of the accumulation signal among the routes (see standard deviations), only for As Cd and Pb there was a significant difference between I7 and the other site typology. It is worth noting that these elements had generally low concentration at 7G, a route embedded in a green area, in which high concentrations of terrigenous elements, as Al and Si were measured.

**Figure 3:**
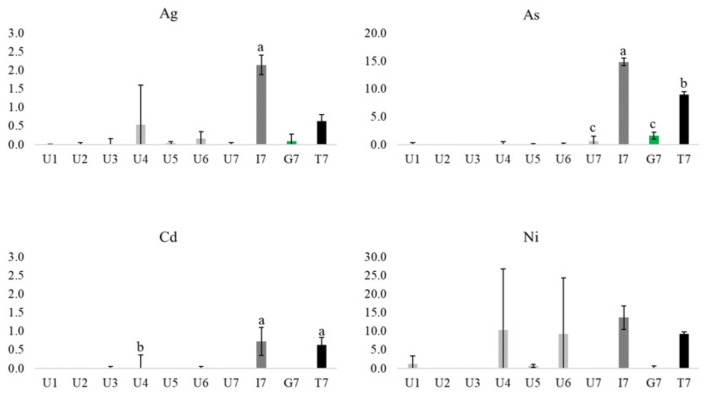

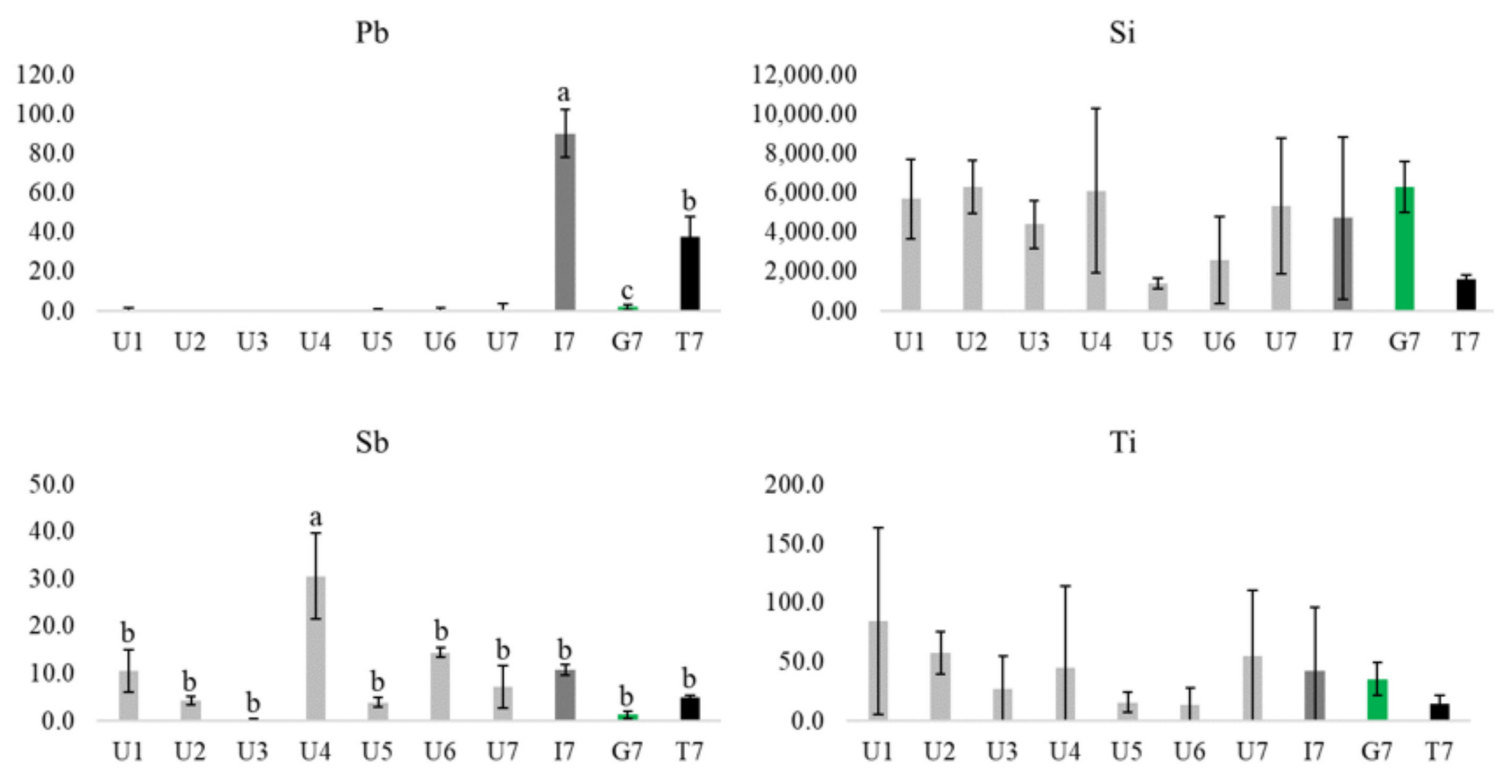
Daily deposition flux (µg m^−2^ d^−1^) for several accumulated elements measured at each route. Different letters indicate significant differences between routes (*p* < 0.05).

The element fluxes (Table S3) reflected the trend of element accumulations along each route. Specifically, they highlighted element daily fluxes rather homogeneous along U routes, except for U4, showing higher fluxes for Na, K, and Sb (the highest, Figure 3), compared to the other U routes. In agreement with elemental contents, the flux was highest in I7 (significantly higher for Ag, As, Cd, and Pb).

### 2.3. Comparison between Moving and Static Moss-Bags

**Figure 4:**
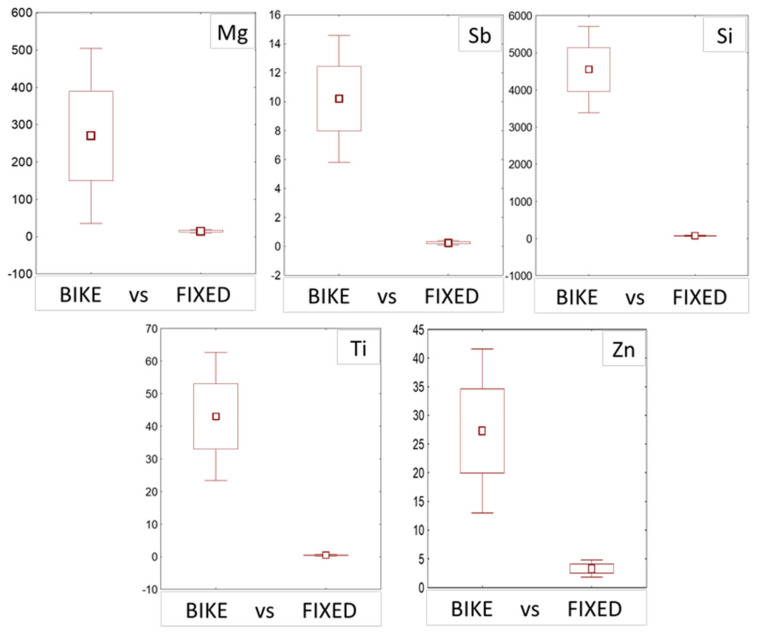
Comparison between fluxes (*y* axis; µg m^−2^ d^−1^) calculated in mosses exposed by bike and in fixed position (*n* = 21), all in the urban environment of Antwerp. Square: Mean; box: Mean ± SE; Whiskers: Mean± 1.96 ∗ SE. For all the comparisons *p* < 0.05, according to the *t*-test for independent samples.

A comparison of the elements accumulated in fixed and bike bags in the urban area of Antwerp (data from Sorrentino et al., 2021), was carried out by averaging data obtained for the seven U routes, and data from bags exposed in fixed position in urban environment (7 exposure sites for a total of 21 moss-bags), for the same element. This comparison highlighted significantly higher fluxes for moss exposed in bikes compared to moss exposed in fixed position, for the elements Mg, Sb, Si, Ti, and Zn (Figure 4). The boxplots also evidenced heterogeneity of replicas in moss samples exposed in bikes.

**Figure 5:**
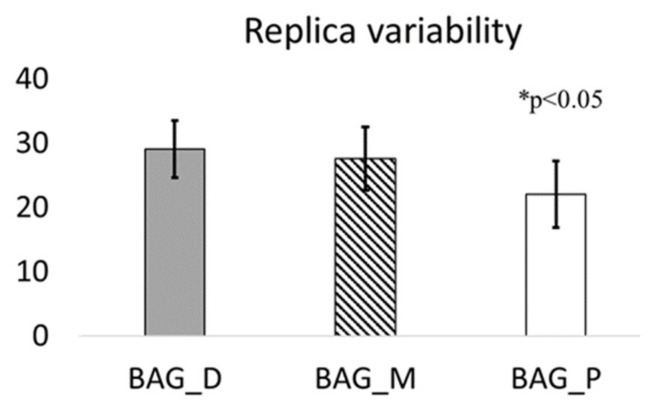
Total element load for each bag position (proximal: P; middle: M; distal: D). Replicas were scored based on their element content between 1 and 3, where 3 represents the highest content and 1 the lowest. The scores of each element were summed to obtain total element load (*y* axis) and the mean values were compared by ANOVA. * *p* < 0.05 according to Tuckey’s post-hoc test.

Both chemical mean content (Table S2) and daily flux of specific elements (Figure 4) evidenced a large variability measured in the three moss replicas, especially for terrigenous elements (Si and Ti). Nevertheless, a decreasing trend of the element content was generally observed passing from the distal to the proximal bag of each triplet (i.e., the proximal bag generally up-took lower amounts) (Figure 5).

### 2.4. Particulate Matter

Average values of daily PM_10_ and PM_2.5_ (expressed in µg m^−3^) recorded along each route were significantly correlated according to Spearman’s rank correlation test (*p* < 0.05). For both PM fractions, the maximum amounts were found along the I route, followed by U, T and G routes. The comparison between PM and element data (i.e., summation of normalized fluxes calculated for each element along each route typology; Table 2) indicated a similar trend between the two data sets (i.e., element fluxes and PM significantly correlated according to Spearman’s rank correlation test, *p* < 0.05).

## 3. Discussion

Our work demonstrated, for the first time, the feasibility of moss-bags to take up airborne elements while carried by bicycles. The ability of the moss-bags to entrap airborne pollutants from mobile sources, for example car or aircraft engines, was already known [22,23]. Also, the idea of mobile monitoring as an innovative tool providing valuable insights into personal exposure to atmospheric pollution is already reported in the literature. For example, Hofman et al. [24] investigated the exposure of a cyclist to black carbon, ultrafine particles, and heavy metals deposited on filters, and found that the exposure was significantly higher while commuting along a vehicle trafficked route compared to a bicycle highway route. However, the different experimental designs, the different methodology, as well as the different analyses carried out do not allow any comparison with the present biomonitoring study.

The results of the present work highlighted a wide variance of the elemental content among replicas; specifically, element concentrations seemed affected by the bag position (with in general a significantly lower element content found in the proximal bag). It is noticeable that the heterogeneity of the chemical data set (Table S1) found its match in the SIRM data, which were on average about 50% higher in the distal bags than in the proximal bags. Although the trend was clear, the differences between bag positions were not significant, however, replicas were always consistent with each other, and between-replica variance was lower than the variance between routes. Thus, despite the different target of the two techniques—ICP-MS aims at quantifying multiple elements, whereas SIRM is sensitive only to the magnetizable fraction of PM, mainly iron oxides like hematite, magnetite and maghemite with incorporated trace elements—both techniques show a clear trend, with higher element fluxes in bags farther away to the handlebar of the bicycle and thus closer to the ground. We applied a system equal to bags exposed in fixed position, i.e., three bags suspended at three different heights along a nylon thread, as in the protocol for exposure at fixed positions. It could be likely that in moving conditions, the proximal bag can shield the other two, due to displacement of air produced while moving, influencing the uptake. Also, the proximal bag could be preserved from the entry of air and related pollutants due to a boundary layer, while the middle and especially the distal bags were moving in the turbulent wake of the proximal bag. As a consequence, the air would pass externally to the proximal bag, without crossing the sample, impeding or reducing the PM interception and entrapment in the proximal bag, while deposition is enhanced in the middle and especially in the distal bag due to higher turbulence. Moreover, the distal bag and, to a lesser extent, the middle bag were closer to the ground, where resuspension and splash from road dust, rich in Fe, Cu, Zn, Mn, Cr, Sb, Sn and Ni [25], can be supposed more relevant than higher up from the ground. To escape replica variability in elemental content, and gain more reliable results, the exposure protocol should be reconsidered. To reduce replica variability, a single larger moss bag, possibly a flat or spherical bag could be more appropriate and moss material could be homogenized before dividing it in three analytical replicas.

A large variability in element content and SIRM was even observed in bags exposed along all U routes, at parity of exposure time (i.e., about 24 h), with particularly high values for U4. It is likely that the very short exposure time, compared to any other biomonitoring study based on fixed bags, could be responsible for the heterogeneity found. The exposure time could play an important role for data homogenization and could represent a key factor for the reliability of the technique [26]; in fact, where the elemental input was stronger, i.e., along the I7 route, a clear signal was observed, specifically for elements of environmental concern, like As, Cd, Ni, Pb and Sb. These elements accumulated in the I7 route and T7 route, the latter of course as the effect of the contribution of the industrial route to the total path. The abundant presence of these metals has been observed in previous biomonitoring studies using leaves sampled near the non-ferrous metallurgical plant around which route I7 ran [27]. The moss-bags in this study seem able to provide information on the pollution level along each route and to emphasize the differences related to the land use. The fact that element concentrations, fluxes and even PM levels provided a similar trend with the highest elemental load and fluxes measured along the I7 route, followed by the U routes, the T7 route and the lowest at the G7 route, indicates that the method is sensitive to the different pollution levels associated to the land uses. A similar differentiation between land uses can be observed based on the SIRM fluxes, in agreement with a biomagnetic study on leaves conducted at urban and industrial sites in Antwerp [28], except for the relatively high SIRM flux along route G7. It is worth noting that Al accumulated only along the G7 route, together with Si and Ti; these elements, regarded as markers of terrigenous inputs, could derive from soil dust resuspension from fields surrounding this path, which possibly also has given rise to higher terrigenous iron oxide input with a higher SIRM flux along G7 as a result. However, further investigations, possibly with higher exposure times and higher number of green and industrial routes, are needed to reach a steady conclusion. It is likely that longer exposure times could provide more accurate data and would reduce the chance of obtaining negative fluxes due to high SIRM and elemental content levels of the unexposed moss.

Although significant differences were observed between routes and elements, we did not present ANOVA results for element contents or accumulation, since they are affected by a different exposure time. Therefore, we prefer to discuss ANOVA analysis on flux data, which are normalized for exposure time (i.e., daily based) for all the elements and SIRM. The formula of element flux, developed in a previous work [29], depends on the specific leaf area, which is considered constant in each species, and on the exposure time. Therefore, this parameter can be profitably used when moss-bags filled with the same species are exposed for different times, even in different conditions (e.g., different land uses). Taking advantage of the above, we compared fluxes calculated for this experimental design, with fluxes calculated for bags filled with *H. cupressiforme* moss and exposed in open air and fixed position in the same study area (i.e., Antwerp city; [17]). The comparison showed that the fluxes of elements in the “mobile bags” were significantly higher than element fluxes measured in “fixed bags” for five elements. This result agrees with literature data; [30], observed that wind could increase the element uptake in moss-bags filled by *Sphagnum palustre* L.; the authors found indeed that metal uptake capacity for As, Cu, Fe, Pb and V in the moss-bags attached to weathervanes was higher than in the moss-bags attached to static poles. In our case, the magnitude of element fluxes while bicycling along the tested routes was several folds higher than for fixed monitoring positions. However, these results should be considered with caution and verified by further experiments over longer periods, based on higher numbers of exposure points and routes.

## 4. Materials and Methods

### 4.1. Moss Material, Study Area and Experimental Design

For the present study, the moss *Hypnum cupressiforme* Hedw. was chosen as plant material, collected in Italy at the protected natural area of Taburno-Camposauro Regional Park (1000 m s.l.m.—Lat. 41.105094° N, Long. 14.593559° E). The moss-bags were prepared as follows: moss was cleaned, washed by deionized water and a Na-EDTA solution, to remove soluble element fraction, oven devitalized and aliquots of 500 mg were used to fill each bag [17,21].

**Figure 6:**
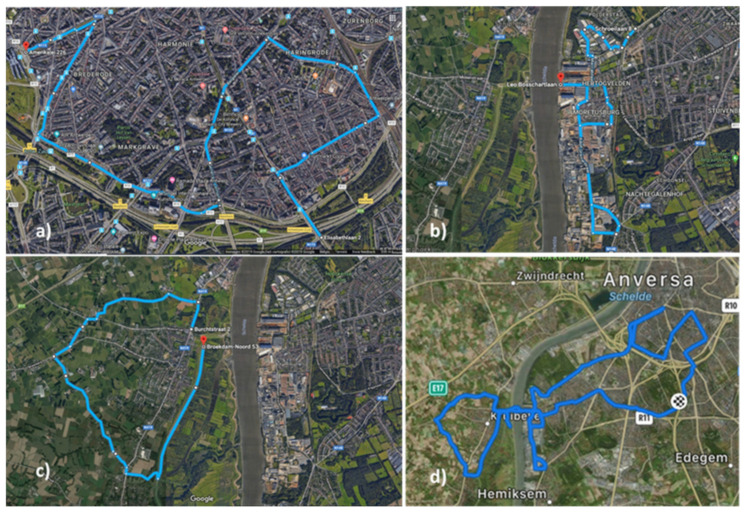
The four routes chosen in the city of Antwerp: (**a**) Urban route; (**b**) Industrial area; (**c**) Green zone; (**d**) Total path.

**Figure 7:**
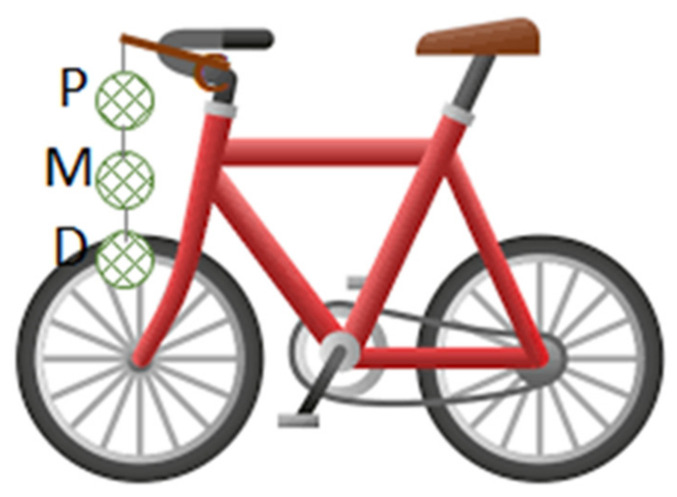
The arrangement of the bicycle hosting moss-bags in triplicate. Bag position: proximal—P; middle—M; distal—D.

In parallel, two experimental designs were carried out in and around the city of Antwerp (51.22° N, 4.40° E) in the Flanders region (northern Belgium) during the period of March-June 2019. The first design involved six volunteer cyclists who carried three moss-bags each on their bicycles (hereafter named U1 to U6) on their daily commute to work through urban areas for 50 days (Table S4). In the second, a single volunteer rode with a bicycle on four routes in selected areas in and around the city for 22 days, carrying three moss-bags for each route. Specifically, the four routes in the city of Antwerp were chosen to cover areas characterized by different land uses (Figure 6): (i) an urban area in the city center of Antwerp along heavily trafficked motorway, ring and national roads and street canyons (U7); (ii) an industrial route in the Hoboken area, in the southern outskirts of Antwerp city, where there is a non-ferrous metallurgical plant and a cement industry (I7); (iii) a green route in Kruibeke, a small community south-east of Antwerp, on the left bank of the Scheldt river through agricultural and green areas (G7); and (iv) a fourth route (T7) was represented by the complete path including the three routes described above and all the connecting streets among them. For each route, the three bags were attached to a stick connected to the handlebar of the bike by means of a nylon rope at three different heights, about 15 cm apart from each other (Figure 7).

The 7th bicycle was equipped with four triplets of bags, each to be exposed during a specific route, and then covered. Each volunteer exposed the moss-bags during the journey, took note of the date and time of the start/end of the exposure, and covered the moss-bags with plastic bags at the end of each route, to avoid further element accumulation while bags stayed in fixed position.

### 4.2. SIRM Analysis

SIRM analysis was carried out according to Sorrentino et al. (2021). After exposure, the moss-bags were dried in an oven (Memmert) at 40 °C for 72 h. Then, whole moss samples (three replicas for each route plus 4 unexposed moss samples as controls) were individually weighed and tightly packed in transparent film to avoid any possible movement during the analysis and placed inside the appropriate plastic container.

All samples were individually magnetized in a direct current (DC) field of 800 mT with a Molspin pulse magnetizer (Molspin Ltd., Witney, Oxfordshire, UK) [11,24]. Immediately after, the remanent magnetization of the samples (A m^−1^) was measured twice using a JR-6 spinner magnetometer (AGICO, Czech Republic) with high sensitivity (2.4·10^−6^ A m^−1^). The instrument was calibrated with a magnetically stable rock supplied as standard by the manufacturer, and its accuracy was checked every ten samples using the same rock specimen as reference sample [31]. The measured values were corrected for the sample holder containing a similar amount of cling film. The methodology is described in detail by [32]. The SIRM was normalized for post-exposure moss dry mass (g) and the size of the plastic container, obtaining mass-normalized SIRM values, expressed in 10^−6^ A m^2^ kg^−1^.

### 4.3. Chemical Analysis

Chemical analysis was performed following Sorrentino et al. (2021). In brief, in each moss sample, the concentration of 28 elements (Al, Ag, As, Be, Ca, Cd, Co, Cr, Cu, Fe, Hg, K, Mg, Mn, Mo, Na, Ni, Pb, Pd, Rb, Sb, Si, Sr, Ti, Tl, U, V, Zn) were measured by HR-ICP-MS or ICP-OES (for Ca, K, Mg and Na). Moss samples were weighed and acid-digested in glass tubes with 2 mL of HNO_3_ and 6 mL of HCl at 100 °C, over-night. After this step, 0.5 mL of H_2_O_2_ was added for digestion into a microwave digester (Discover SP-D, CEM). Digestion was carried out at 180 °C, ramp time 5 min and hold time 5 min. The digested solution was transferred to plastic tubes and deionized water was added until 40 mL. Elements were considered accumulated when their post-exposure concentration was higher than the pre-exposure concentration + 2 ∗ SD [33,34]. For quality control of the analysis, certified reference plant material (Certified Reference Material BCR^®^-679, white cabbage) was analyzed in parallel with samples. For the elements indicated in the certified material, the percentages of recovery were in the range of acceptability, specifically from 80% to 105% (Ca 99.63%, Cd 80.15%, Cr 84.80%, Cu 91.55%, Fe 95.13%, Mg 89.28%, Mn 92%, Ni 99.35%, Sr 85.69%, Zn 88.49%), and below 80% for As (60.42%). The data were normalized considering the dilution factor and the mass of each sample.

### 4.4. PM Sampling

The bicycle of volunteer 7 (who moved along the 7U, 7G, 7I and 7T routes) was —besides with moss-bags — equipped with a PM sampler SDS01 for PM_2.5_ and PM_10_ (Nova Fitness Co., Ltd. Jinan, Shandong Province, China) with the inlet attached at the same height as the proximal moss bag. This sampler measured every minute the PM concentration during each route. Based on the annotation of the starting and ending times of each route, the PM values measured every day could be assigned to a specific route.

### 4.5. Data Analyses

Raw data elaborations and basic statistics were processed by Microsoft Excel. The threshold to determine element accumulation in each sample was fixed in the limit of quantification of the technique (LOQT), calculated from the initial concentrations as follows: xCi + 2sCi, where xCi is the mean value of the initial concentration in unexposed moss samples (*n* = 4) for each element determined, and sCi is the corresponding standard deviation [33,34].

The daily flux for each element was calculated according to Capozzi et al., 2020, using the following formula:

ΘDF = [M]acc∗(2∗SLA∗d)^−1^

where ΘDF is the deposition flux [µg m^−2^ d^−1^]; [M]acc is the concentration of each element (expressed as µg kg^−1^) accumulated during the exposure period or the accumulated SIRM (expressed as µA m^2^ kg^−1^ and calculated as the SIRM of exposed moss minus the mean SIRM of the unexposed moss), obtained by subtracting the pollutant concentration found in pre-exposure moss from that measured after the exposure; SLA = specific leaf area, i.e., fresh leaf area (m^2^)/dry weight (kg); d is the exposure time expressed in days.

Spearman Rank Order Correlation was performed to assess the correlation between the element accumulation and the SIRM signal. Also, an ANOVA was carried out to check for significant differences in elemental fluxes and SRIM data (these latter normalized for the exposure time) among all the routes.

The *t*-test for independent samples was used to compare element accumulation by bike vs moss-bags exposed outdoors fixed to balconies [17]. The fixed moss-bags were selected in the same study area and among those closest to the routes of the present study, for a total of seven urban sites and seven routes (*n* = 21, both for fixed and for mobile bags). For the multiple comparisons, we used false discovery rate (FDR) adjusted *p*-values using Benjamini-Hochberg’s correction.

To compare the PM values to the element data, elemental fluxes were normalized to each maximum value (i.e., between 0 and 1) to equally weigh all the elements. Then, normalized fluxes of each element were summed to obtain total normalized flux calculated at each route to be compared to the averaged PM_10_ and PM_2.5_ amount. To test the significance among replicas (proximal, middle, and distal bags) exposed along the different routes the elemental contents were scored between 1 to 3, according to their lower-higher element content measured in each sample, and summed in each bag class (i.e., proximal, middle, and distal) to express the level of total element load for each class. The difference between bag classes was tested by ANOVA.

## 5. Conclusions

This work encourages further studies based on the moss-bag technique for air biomonitoring with mobile systems (bicycles or even electric bicycles/motorcycles) since it highlights the capacity of this device to uptake airborne pollutants even when exposed while travelling. However, some critical issues have arisen that lead to reconsidering the exposure conditions to obtain more reliable results. Specifically, the importance of the exposure times and the possibility to adopt analytical replicas instead of experimental ones, could be studied in depth, with the aim to understand the mechanisms under the high replica variability in element contents observed in this work. Although different pollution levels were highlighted among the land uses investigated in this pilot work, a finetuning of the methodology could lead to a better sensitivity in case of a more continuous pollution gradient. Enviromagnetic analysis of exposed moss samples, represents a fast and robust methodology to gain information about airborne element pollution, even in moving systems. In general, both SIRM and element fluxes of exposed mosses, as well as atmospheric PM concentrations indicated the industrial route as the most polluted and the green route as the area having the lowest level of air pollution. Further, the comparison between pollution level measured in static and moving bags in terms of element fluxes, indicates that the magnitude of the signal is significantly higher in mobile systems than in fixed positions, at parity of land use. Mobile biomonitoring by SIRM and ICP-MS of moss transplants seems a promising approach that could provide new outcomes, or a broader application, in the evaluation of the total personal exposure to air pollution.

## Figures and Tables

**Table 1:** Correlation coefficient (R), *t*-value and *p*-value of the relationship between SIRM and elements of the exposed moss.

	R	T (N-2)	*p*
Ag	0.609	4.071	<0.001
As	0.582	3.789	<0.001
Cd	0.607	4.038	<0.001
Co	0.509	3.131	0.004
Hg	0.494	3.007	0.005
Ni	0.451	2.673	0.012
Pb	0.638	4.392	<0.001

**Table 2:** Summation of normalized fluxes, PM_10_ and PM_2.5_ average concentrations (µg m^−3^).

	∑ Normalized Fluxes	PM_10_	PM_2.5_
U7	6.8	23.3	9.7
I7	10.5	25.9	9.9
G7	2.3	20.3	3.1
T7	5.7	21.9	9.1

## Data Availability

All data are reported in the text and in Supplementary Materials.

## Supplementary Materials

The following are available online at https://www.mdpi.com/article/10.3390/plants10112384/s1, Table S1. Mean element content (mg kg^−1^ DW) in pre-exposed moss (BL) and standard deviation (SD); LOQT (limit of quantification of the technique; see the text for details); element content in each exposed moss sample. Concentrations higher than LOQT are marked in light pink. All element concentrations and LOQT are expressed as mg kg^−1^ DW. Table S2. Mean element concentrations and SD (*n* = 3, mg kg^−1^) measured for each route at the end of experiment; the element content and the standard deviation of the pre-exposed moss is indicated as BL (baseline). BDL: below detection limit. Table S3. Daily deposition flux (µg m^−2^ d^−1^) of accumulated elements along each route. Different letters indicate significant differences according to Tukey’s post-hoc test (*p* < 0.05). Table S4. For each route travelled by the six volunteers (U1 to U6), the total distance travelled (km) with moss exposed on their bikes, the total exposure time (h) of the moss bags, and the average speed (km/h).
