# Supplementary material for: Mobile Biomonitoring of Atmospheric Pollution: A New Perspective for the Moss-Bag Approach
Source: Plants (Basel). 2021 Nov 5;10(11):2384. doi: 10.3390/plants10112384 (PMC8621684; doi:10.3390/plants10112384)
Supplement: Supplementary file 1 [file plants-10-02384-s001.zip › Table S1.pdf]

Table S1. Mean element content (mg kg^{-1} DW) in pre-exposed moss (BL) and standard deviation (SD); LOQT (limit of quantification of the technique; see the text for details); element content in each exposed moss sample. Concentrations higher than LOQT are marked in light pink. All element concentrations and LOQT are expressed as mg kg_{-1} DW.

	Ag	Al	As	Ca	Cd	Co	Cr	Hg	K	Mg	Mo	Na	Ni	Pb	Si	Sb	Ti	Zn
**BL** mean (n=4)	0.045	5817	0.873	2097	0.065	0.823	2.380	0.117	1849	887	0.293	8258	2.40	4.37	1922	0.275	23.1	23.8
SD	0.015	481	0.037	284	0.015	0.043	0.048	0.005	70	45	0.065	349	0.09	0.21	54	0.067	0.6	1.8
**LOQT**	0.076	6779	0.948	2665	0.096	0.909	2.477	0.127	1988	977	0.423	8956	2.58	4.80	2030	0.408	24.3	27.5
U1	0.047	5422	0.790	1783	0.043	0.605	2.397	0.087	1994	809	0.446	8790	2.86	4.57	2575	1.603	30.2	25.7
U1	0.034	5303	0.918	3267	0.057	0.458	1.495	0.100	2104	904	0.342	8729	2.60	4.27	3049	2.465	47.8	27.3
U1	0.023	4915	0.884	1800	0.011	0.571	1.727	0.087	1856	812	0.193	8553	2.26	3.74	2544	1.228	27.1	24.3
U2	0.053	4855	0.661	1838	0.021	0.500	1.649	0.086	1836	829	0.187	8736	2.10	3.71	2532	0.887	30.4	23.8
U2	0.030	5445	0.787	2567	0.027	0.581	1.765	0.084	1896	883	0.200	8407	2.06	3.89	2861	0.874	32.4	24.3
U2	0.042	5855	0.829	1826	0.048	0.656	1.799	0.097	1836	848	0.229	8228	2.33	4.12	2712	0.687	28.0	25.1
U3	0.038	5988	0.816	1564	0.077	0.671	2.150	0.104	1664	771	0.274	7519	2.38	3.95	2515	0.333	25.1	29.2
U3	0.072	5622	0.836	2405	0.035	0.618	1.494	0.092	1748	810	0.226	7659	2.10	4.09	2721	0.330	31.5	22.9
U3	0.032	5243	0.766	1656	0.023	0.695	1.952	0.101	1753	819	0.228	8230	2.28	3.78	2387	0.271	24.2	22.5
U4	0.170	5221	0.930	2328	0.096	0.817	2.251	0.115	4174	1037	0.327	11329	4.46	4.42	2213	2.593	24.5	34.0
U4	0.046	4744	0.731	2008	0.013	0.680	1.826	0.099	2422	969	0.266	9118	2.59	3.78	2690	2.967	31.8	27.8
U4	0.034	6097	0.740	1762	0.032	0.667	1.967	0.100	1962	844	0.227	8578	2.33	3.87	2152	1.730	22.6	26.1
U5	0.086	4739	0.918	2509	0.020	0.728	2.497	0.095	2194	1074	0.457	9041	2.62	4.88	2691	2.978	31.0	40.6
U5	0.078	4115	0.629	2416	0.039	0.487	2.438	0.081	1829	938	0.360	7632	2.98	4.06	2785	2.076	35.9	42.0
U5	0.044	5466	0.869	1924	0.033	0.731	2.272	0.101	1930	909	0.282	8276	2.69	4.09	2511	1.948	27.2	32.8
U6	0.094	5922	0.916	2165	0.044	0.815	3.812	0.114	2268	1026	0.448	9425	5.82	4.62	2029	1.968	23.5	33.0
U6	0.049	4803	0.779	1853	0.074	0.644	1.713	0.101	1991	838	0.267	8474	2.37	4.07	2349	2.179	24.2	28.4
U6	0.054	5064	0.773	1954	0.026	0.645	2.192	0.099	1952	905	0.229	8598	2.59	4.04	2480	2.228	27.0	28.0
U7	0.036	4582	1.088	2054	0.042	0.577	1.708	0.091	1834	859	0.173	8099	2.29	4.91	3091	1.734	38.1	26.2
U7	0.047	4077	0.896	1773	0.036	0.598	1.542	0.101	1854	836	0.198	8141	2.31	4.10	2445	1.190	26.7	23.1
U7	0.051	6737	0.860	2189	0.057	0.713	2.067	0.113	2032	951	0.252	8860	2.37	4.08	2256	0.614	25.5	22.8
I7	0.334	4102	2.619	1260	0.199	0.468	1.226	0.094	1386	565	0.156	6220	3.61	14.46	1921	1.666	20.8	19.1
I7	0.277	5901	2.710	1883	0.113	0.821	1.597	0.126	2062	1007	0.225	8556	4.36	16.63	2727	1.565	32.5	26.4
I7	0.287	8082	2.555	1867	0.141	0.916	1.592	0.118	2067	1003	0.217	8367	4.09	13.98	2791	1.410	31.1	25.8
G7	0.042	7126	1.075	1907	0.060	0.821	1.658	0.112	1933	912	0.253	7774	2.50	4.69	2477	0.533	26.5	23.0
G7	0.081	6725	1.122	1774	0.047	0.701	1.630	0.091	1894	911	0.207	8127	2.26	4.70	2762	0.386	29.1	22.1
G7	0.046	4765	0.977	1679	0.061	0.601	1.376	0.096	1858	864	0.184	8482	2.12	4.40	2700	0.352	26.1	21.9
T7	0.431	5385	5.285	2012	0.464	0.855	1.940	0.151	1931	909	0.296	8163	6.62	27.15	2579	2.792	27.6	32.6
T7	0.296	6205	4.974	1980	0.294	0.919	1.933	0.139	1767	869	0.305	7344	6.97	19.75	2715	2.580	28.8	30.7
T7	0.289	5156	4.743	1956	0.317	0.731	1.810	0.128	2047	943	0.230	8918	6.40	18.80	2737	2.395	33.3	31.8
