# Supplementary material for: Mobile Biomonitoring of Atmospheric Pollution: A New Perspective for the Moss-Bag Approach
Source: Plants (Basel). 2021 Nov 5;10(11):2384. doi: 10.3390/plants10112384 (PMC8621684; doi:10.3390/plants10112384)
Supplement: Supplementary file 1 [file plants-10-02384-s001.zip › Table S2.pdf]

Table S2. Mean element concentrations and SD (n=3, mg kg^{-1}) measured for each route at the end of experiment; the element content and the standard deviation of the pre-exposed moss is indicated as BL (baseline). BDL: below detection limit.

	**Ag**	**Al**	**As**	**Be**	**Ca**	**Cd**	**Co**	**Cr**	**Cu**	**Fe**	**Hg**	**K**	**Mg**	**Mn**
**BL**	0.04±0.01	5817±480	0.87±0.03	0.46±0.1	2096±283	0.06±0.01	0.82±0.04	2.38±0.04	13.6±6.02	2150±59.4	0.11±0.00	1848±69.6	886±44.9	55.6±0.85
**U1**	0.03±0.01	5213±264	0.86±0.06	0.2±0.11	2283±851	0.03±0.02	0.54±0.07	1.87±0.46	11.4±0.42	1567±95.5	0.09±0.00	1984±124	841±54	42.9±1.89
**U2**	0.04±0.01	5385±502	0.75±0.08	0.11±0.06	2076±424	0.03±0.01	0.57±0.07	1.73±0.07	10.1±0.13	1636±134	0.08±0.00	1855±34.4	853±27.3	42.8±2.7
**U3**	0.04±0.02	5617±372	0.8±0.03	0.22±0.02	1875±461	0.04±0.02	0.66±0.03	1.86±0.33	10.1±0.38	1767±213	0.09±0.00	1722±49.9	799±25.6	46.3±5.63
**U4**	0.08±0.07	5353±686	0.8±0.11	0.26±0.05	2032±283	0.04±0.04	0.72±0.08	2.01±0.21	15.3±5.85	1814±84.6	0.1±0.00	2852±1167	949±98.1	47.1±1.41
**U5**	0.06±0.02	4773±676	0.8±0.15	0.18±0.09	2282±314	0.03±0.00	0.64±0.14	2.4±0.11	21±2.71	1849±253	0.09±0.01	1984±188	973±88	46.4±4.87
**U6**	0.06±0.02	5262±585	0.82±0.08	0.2±0.03	1990±158	0.04±0.02	0.7±0.09	2.57±1.1	12.6±1.59	1900±300	0.1±0.00	2070±172	923±95.4	49.6±3.89
**U7**	0.04±0.00	5132±1412	0.94±0.12	0.18±0.11	2005±211	0.04±0.01	0.62±0.07	1.77±0.26	10.4±0.47	1746±176	0.1±0.01	1906±109	881±61	44.1±2.65
**I7**	0.29±0.03	6028±1993	2.62±0.07	0.21±0.08	1670±355	0.15±0.04	0.73±0.23	1.47±0.21	15.8±1.29	1739±463	0.11±0.01	1838±391	858±253	42.1±11.1
**G7**	0.05±0.02	6205±1263	1.05±0.07	0.18±0.02	1786±114	0.05±0.00	0.7±0.11	1.55±0.15	9.78±0.26	1719±200	0.09±0.01	1894±37.4	895±27.4	43.1±4.19
**T7**	0.33±0.07	5582±551	5±0.27	0.15±0.00	1982±28.3	0.35±0.09	0.83±0.09	1.89±0.07	22.8±1.31	1921±72.4	0.13±0.01	1914±140	907±37	49.7±3.19

	**Mo**	**Na**	**Ni**	**Pd**	**Pb**	**Rb**	**Si**	**Sb**	**Sr**	**Ti**	**Tl**	**U**	**V**	**Zn**
**BL**	0.29±0.06	8257±349	2.4±0.08	BDL	4.36±0.21	6.93±0.44	1922±53.8	0.27±0.06	17.7±0.87	23.1±0.56	0.18±0.04	0.27±0.01	7.28±0.28	23.7±1.83
**U1**	0.32±0.12	8690±122	2.57±0.3	BDL	4.19±0.42	3.24±0.29	2722±282	1.76±0.63	15.5±3.18	35±11.10	0.17±0.04	0.22±0.01	4.05±0.87	25.7±1.52
**U2**	0.2±0.02	8456±257	2.16±0.14	BDL	3.9±0.2	1.86±0.98	2701±164	0.81±0.11	12.9±1.29	30.2±2.21	0.16±0.00	0.22±0.03	4.53±0.33	24.4±0.64
**U3**	0.24±0.02	7802±376	2.25±0.14	BDL	3.93±0.15	2.56±1.09	2541±168	0.31±0.03	12.2±0.92	26.9±3.96	0.17±0.01	0.24±0.00	5.1±0.85	24.8±3.72
**U4**	0.27±0.05	9674±1457	3.12±1.15	BDL	4.02±0.34	3.11±1.34	2351±294	2.42±0.63	11.5±0.85	26.2±4.86	0.17±0.01	0.24±0.00	5.33±0.52	29.2±4.16
**U5**	0.36±0.08	8316±705	2.76±0.19	BDL	4.34±0.46	2.07±0.36	2662±139	2.33±0.56	10.9±1.00	31.3±4.37	0.16±0.02	0.21±0.02	4.99±1.47	38.4±4.96
**U6**	0.31±0.11	8832±516	3.59±1.93	BDL	4.24±0.32	2.61±0.96	2254±284	2.12±0.13	10.7±0.68	24.8±1.83	0.17±0.00	0.23±0.00	5.43±1.23	29.7±2.78
**U7**	0.2±0.04	8366±427	2.32±0.04	BDL	4.36±0.47	1.76±0.77	2597±437	1.17±0.56	10.7±1.04	30.1±6.98	0.16±0.01	0.22±0.02	4.15±1.01	24±1.86
**I7**	0.19±0.03	7714±1297	4.02±0.37	BDL	15±1.41	1.69±1.06	2479±484	1.54±0.12	10.4±2.9	28.1±6.37	0.17±0.03	0.23±0.06	4.13±1.05	23.8±4.06
**G7**	0.21±0.03	8127±353	2.29±0.19	BDL	4.59±0.17	2.48±0.81	2646±150	0.42±0.09	12±1.25	27.2±1.64	0.18±0.02	0.23±0.01	4.12±0.57	22.3±0.57
**T7**	0.27±0.04	8141±787	6.66±0.28	BDL	21.9±4.57	2.01±0.75	2676±85.7	2.58±0.19	13±1.21	29.8±3.00	0.19±0.01	0.25±0.01	4.68±0.39	31.6±0.92
