# Supplementary material for: Mobile Biomonitoring of Atmospheric Pollution: A New Perspective for the Moss-Bag Approach
Source: Plants (Basel). 2021 Nov 5;10(11):2384. doi: 10.3390/plants10112384 (PMC8621684; doi:10.3390/plants10112384)
Supplement: Supplementary file 1 [file plants-10-02384-s001.zip › Table S3.pdf]

Table S3. Daily deposition flux (µg m^{-2} d^{-1}) of accumulated elements along each route. Different letters indicate significant differences according to Tukey's post-hoc test ($p<0.05$)

	Ag	As	Cd	Cr	Hg	K	Na	Ni	Pb	Si	Sb	Ti	Zn
**U1**	0.009e	0.194d	0	0.122b	0	967c	3078b	2.351c	1.436c	5691b	10.60b	84.62a	14.14c
**U2**	0.059d	0	0	0	0	379d	2535c	0	0	6297b	4.37c	57.61b	5.15d
**U3**	0.190c	0	0.083d	0	0	0	0	0	0	4401c	0.40d	26.86c	38.14b
**U4**	0.886b	0.798c	0.438c	0	0	14278a	20154a	15.952a	0.730c	6106b	30.65a	71.43a	77.87a
**U5**	0.069d	0.084d	0	0.166b	0	405d	762	0.689d	0.967c	1408d	3.92c	15.70d	27.95b
**U6**	0.160c	0.333d	0.067d	11.192a	0	1732b	4489b	14.087a	1.953c	2599d	14.46b	13.60d	46.94b
**U7**	0.028e	0.937c	0	0	0	743c	4759b	0	4.272c	5333c	7.15b	55.00b	18.78c
**I7**	2.147a	14.855a	0.727a	0	0.039b	1826b	1726c	13.707a	90.200a	7083a	10.77b	73.45a	19.89c
**G7**	0.155c	1.603c	0	0	0	401d	1946c	0.823d	1.963c	6296b	1.29c	35.51c	0
**T7**	0.635b	8.948b	0.636b	0	0.048a	304d	1432c	9.236b	38.007b	1636d	5.02c	14.65d	17.13c
