# Supplementary material for: Mobile Biomonitoring of Atmospheric Pollution: A New Perspective for the Moss-Bag Approach
Source: Plants (Basel). 2021 Nov 5;10(11):2384. doi: 10.3390/plants10112384 (PMC8621684; doi:10.3390/plants10112384)
Supplement: Supplementary file 1 [file plants-10-02384-s001.zip › Table S4.pdf]

Table S4. For each route travelled by the six volunteers (U1 to U6), the total distance travelled (km) with moss exposed on their bikes, the total exposure time (h) of the moss bags, and the average speed (km/h).

	Route (km)	Exposure time (h)	Average speed (km/h)
**U1**	300	25	12
**U2**	297	22	13.5
**U3**	310	25	12.4
**U4**	180	13	13.8
**U5**	1700	93	18.3
**U6**	340	22	15.4
**U7**	352	22	16
**I7**	330	21	15.7
**G7**	308	20	15.4
**T7**	1100	82	13.4
